# Structural Coloration and Carotenoids Together Create the Vibrant Colors of Peafowl Feathers

**DOI:** 10.3390/ani16060903

**Published:** 2026-03-13

**Authors:** Gang Wang, Xinye Zhang, Xiurong Zhao, Xufang Ren, Zhonghua Ning, Lujiang Qu

**Affiliations:** 1 College of Animal Science and Technology, Tarim University, Alar 843300, China; wanggang@cau.edu.cn; 2 College of Animal Science and Technology, China Agricultural University, Beijing 100107, China; xinye_leaf@163.com (X.Z.); zxiurong_feign@163.com (X.Z.); rxf1828@163.com (X.R.); ningzhh@cau.edu.cn (Z.N.)

**Keywords:** peafowl, lutein, feather color, *ASIP*, *GSTA2*

## Abstract

Previous studies have considered the iridescent feathers of the peafowl as a classic example of structural coloration. The structural color is primarily attributed to a two-dimensional (2D) photonic crystal structure composed of melanin rods and air channels embedded in a keratin matrix. While previous optical models have successfully explained spectral tuning via geometric parameters such as lattice constants and cortex thickness, the potential contribution of auxiliary pigments to these complex hues has been largely overlooked. In this study, we combined high-sensitivity UPLC-MS and transcriptome analysis to elucidate the biochemical and genetic mechanisms underlying peafowl coloration. We identified trace amounts of the Xanthophyll lutein (one of the carotenoids) in iridescent train feathers, challenging the purely structural paradigm. Transcriptome analysis revealed significant differences in the expression of the melanin-related gene *ASIP* between iridescent and non-iridescent feather follicles. Furthermore, we observed significant expression differences in the carotenoid deposition-related gene *GSTA2*, correlating with the presence of lutein in iridescent regions. We conclude that while melanin provides the structural foundation for iridescence, lutein acts as an indispensable conditional modulator. The coordinated differential expression of melanin synthesis (*ASIP*) and carotenoid deposition (*GSTA2)* genes constitutes the genetic basis for the vibrant iridescent coloration of peafowl feathers.

## Simple Summary

The vibrant, iridescent feather of the peafowl is a classic example of nature’s optical engineering. While it is well-known that these colors arise from a physical structure of melanin rods, the role of other chemical pigments has remained unclear. In this study, we identified the presence of the Xanthophyll lutein in peafowl feathers using high-sensitivity UPLC-MS/MS. Our findings suggest that iridescence is not purely structural; instead, it is a dual-mechanism system where melanin provides the physical foundation for light scattering, while Xanthophyll lutein acts as a chemical filter to fine-tune the final color. Genetic analysis revealed that the genes *ASIP* (related to melanin) and *GSTA2* (related to carotenoid deposition) are expressed differently in iridescent versus non-iridescent feathers. This indicates that the precise coordination of these two genes forms the hereditary basis for the peafowl’s iconic ocelli patterns.

## 1. Introduction

The iridescent feathers of the peafowl are considered a classic example of animal structural color [1,2]. During the mating season, male peafowls spread their train feathers to attract females [3]. Their blue, green and brown richly patterned train feathers are a great way to attract females. Through their colorful feathers, peafowls can convey important information about an individual’s identity, health, and social superiority [4,5,6,7]. Previous studies have shown that the iridescent feathers of the peafowl contain biophotonic structures consisting of two-dimensionally ordered lattices of cylindrical melanosomes and air channels embedded in keratin. And these biophotonic structures have slight variations in different feather regions [1,8,9].

However, whether peafowl feathers contain fat-soluble pigments other than melanin as an important factor has been ignored in the study of male peafowl feather color [1]. Carotenoids are the most common class of fat-soluble pigments in birds, and about 30 different carotenoids have been identified in hundreds of bird species [10]. It is characterized by a central chain with conjugated double bonds, which absorbs short wavelengths (blue and violet) of visible light and reflects longer wavelengths of light, appearing red, orange or yellow [11,12]. However, when carotenoids form complexes with structural proteins, these colors can turn into blue, purple or green [13]. Due to the inability of birds to synthesize de novo carotenoids, carotenoid coloration involves four major physiological processes, including carotenoid uptake in the digestive system, transport in the circulatory system, metabolism in epithelial tissues or liver, and deposition in pigmented tissues on the body surface [14,15,16]. Genetic variation in any of these processes may have a significant impact on the phenotype [17]. It remains unknown whether other colors of peafowl feathers expect blue, such as yellow-green, brown, and purple, are affected by carotenoids, and their gene expression patterns also need to be explored.

In this study, we confirmed the presence of the xanthophyll lutein in peafowl feathers by combining ultra-high-performance liquid chromatography (UHPLC) in combination with mass spectrometry (UHPLC-MS) analyses of male peafowl feathers. In addition, we also analyzed the differential expression of carotenoid-related genes in feathers of different colors by sequencing the transcriptomes of feather follicle tissues of different colors.

## 2. Materials and Methods

### 2.1. Peafowl Feathers and Feather Follicle Samples

Feather and feather follicle samples were obtained from two species: three adult male Indian peafowl (*Pavo cristatus*) from Beijing Agricultural College and three adult male green peafowl (*Pavo muticus*) from Qinhuangdao Wildlife Park, Hebei Province. Samples were collected from three anatomical regions: the rump, mantle, and breast. For the train feathers (rump), the ocelli region was further micro-dissected into four color-specific zones: Rump_G (green), Rump_Br (brown), Rump_B (blue), and Rump_P (purple). Simultaneously, feather follicle tissues were harvested from the same anatomical sites for transcriptomic analysis. A total of 9 samples were collected from the Indian peafowl (3 locations × 3 individuals) and 18 samples from the green peafowl (3 locations × 3 individuals × 2 replicates) (Table S1). While some of the feather and tissue samples were consumed during the RNA extraction and experimental procedures, the remaining samples are deposited at China Agricultural University. All sampling procedures were conducted with formal institutional permission from the respective facilities. Sample collection was performed by a licensed professional veterinarian to ensure minimal stress and no harm to the birds. All procedures complied with institutional and national guidelines for the care and use of animals in research.

### 2.2. Ultra-High-Performance Liquid Chromatography and Mass Spectrometry of Peafowl Feathers

Carotenoid composition and concentration in the feathers were determined using Ultra-High-Performance Liquid Chromatography-Tandem Mass Spectrometry (UHPLC–MS/MS). Approximately 0.1 g of the feather sample was homogenized in 1 mL of methanol (containing 0.1% BHT), refrigerated overnight at 4 °C, and centrifuged (3000 rpm, 5 min). The supernatant was reserved, and the pellet underwent two additional extractions with n-hexane (1 mL). Combined extracts were evaporated to dryness under nitrogen gas at 30 °C and reconstituted in 0.2 mL of methanol.

Chromatographic analysis was performed on a Thermo U3000 UPLC system (Thermo Fisher Scientific, Waltham, MA, USA) with a YMC Carotenoid S-3 μm column (150 × 4.6 mm; YMC Co., Ltd., Kyoto, Japan) maintained at 40 °C. The mobile phase comprised methanol (A) and a ternary mixture (methanol:MTBE:water = 20:75:5) (B) using a 30 min gradient program. Mass Spectrometry: Detection was conducted using an Orbitrap Exploris 120 mass spectrometer (Thermo Fisher Scientific, Waltham, MA, USA). Components were identified by comparing retention times, specific absorbance wavelengths, and MS data against a 9-carotenoid standard (Sigma-Aldrich, St. Louis, MO, USA) and published literature [18,19]. Analysis was performed on both whole train feathers and the four color-separated ocelli regions. Significant differences were determined by a two-tailed Student’s *t*-test assuming equal variances.

### 2.3. Improving the Annotation Completeness of the Green Peafowl Genome

To enhance the annotation completeness of the green peafowl genome, an integrative pipeline was employed. Repetitive elements were identified and masked using RepeatModeler2 and RepeatMasker (RepBase). Ab initio predictions were generated using BRAKER3 (integrating AUGUSTUS and GeneMark-ET with homology-trained HMMs) and the deep learning framework Helixer to model gene structures [20,21]. Homology-based annotations were derived from miniprot alignments against avian proteomes [22]. Transcriptomic evidence was integrated by mapping new feather follicle RNA-seq reads using HISAT2, followed by assembly with StringTie and ORF prediction via TransDecoder. Gene prediction results were weighted based on evidence confidence (10 for transcriptome evidence, 5 for homology evidence, and 1 for ab initio predictions) and integrated into a consensus gene set using EvidenceModeler (V.3.0.0) [23].

### 2.4. Feather Follicle Tissues Transcriptome Sequencing and Analysis

Fresh feather follicle tissues were placed in liquid nitrogen and ground into powder using a pre-chilled mortar. Total RNA was extracted from liquid nitrogen-ground feather follicles using the TRIzol method. RNA purity (A260/A280 ≈ 2.0) was assessed using spectrophotometry. cDNA libraries (150 bp inserts) were constructed and sequenced on the Illumina HiSeq 2000 platform (BGI Technologies, Shenzhen, China). For RNA-seq data analysis, the clean paired-end reads were mapped to the peafowl reference genome (WP-1) using the HISAT2 (V.2.1.0) software after quality control (Table S2) [3,24]. Transcripts were assembled and quantified with the StringTie (V.3.0.3) [25]. The GFFcompare (V.0.12.10) was used to compare the alternative transcripts among individuals [26]. The differential expression analysis was performed using the DESeq2 package to target the mechanisms underlying their distinct iridescent characteristics [27]. Ultimately, we enriched candidate genes using the online website KOBAS (V.3.0.0) [28], so as to grasp the functions of selection genes and differential expression genes (DEGs). Furthermore, we compared the differential expression of melanin and carotenoid-related genes in different feather follicle tissues. Significant differences were determined by *t*-tests, and *p*-values were adjusted for multiple comparisons using the Benjamini–Hochberg (BH) method to control the false discovery rate (FDR) [29].

## 3. Results

### 3.1. Xanthophyll Lutein (One of Carotenoids) in Peafowl Feathers

**Figure 1:**
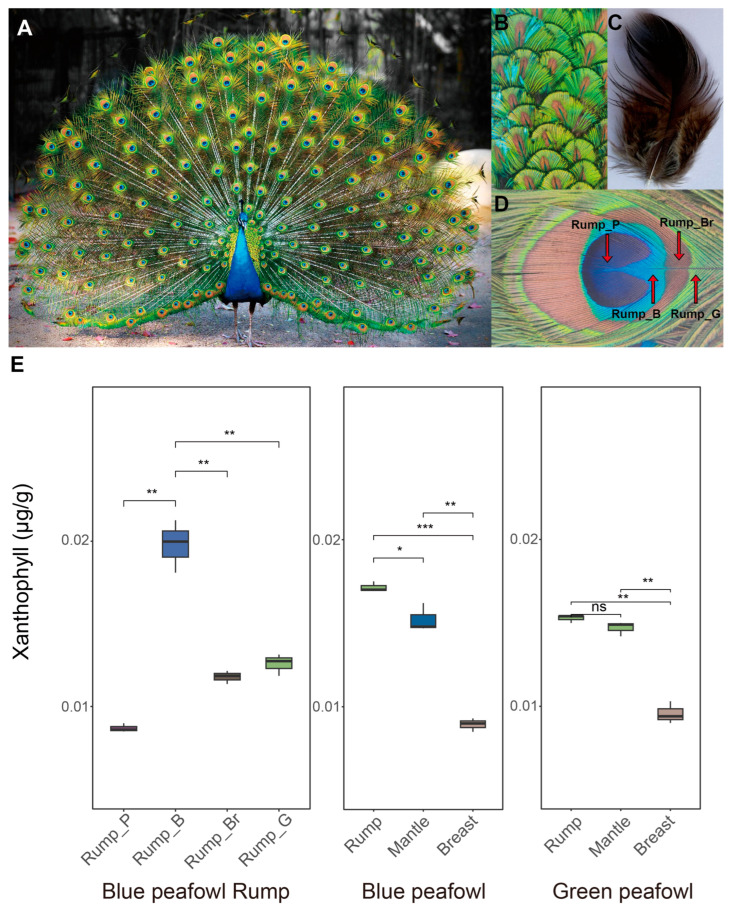
Blue peafowl feathers and Xanthophyll lutein content. (**A**) Photo of an adult male blue peafowl. (**B**) The striking blue back feathers of the blue peafowl. (**C**) The non-iridescent gray breast feathers of the blue peafowl. (**D**) The blue peafowl has iridescent train feathers growing from its rump. The ocelli on the iridescent feathers were divided into four regions based on their different colors (Rump_G, Rump_Br, Rump_B, and Rump_P). (**E**) Xanthophyll lutein content in feathers from different parts of the blue peafowl and green peafowl. Significant differences are indicated by asterisks (^ns^ *p* > 0.05, * *p* < 0.05, ** *p* < 0.01, *** *p* < 0.001) as determined by *t*-tests.

Male peafowls exhibit diverse feather coloration, with green feathers on their mantles and gray feathers on their bellies. Their train feathers growing on the rump are the most colorful and longest, with vibrant ocelli patterns at the tips. The iridescent colors of male peafowl feathers are thought to be structural colors composed of melanosomes and air channels embedded in keratin [1,2,8,9], but previous studies seem to have overlooked the possibility that carotenoids might be involved in the formation of these iridescent feathers (Figure 1A–D).

We used ultra-high-performance liquid chromatography-mass spectrometry (UHPLC-MS) and carotenoid standards to analyze the carotenoid composition of three different parts (rump, mantle, and breast) of blue peafowls and green peafowls, as well as different colored regions of the train feather ocelli growing on the rump. By comparing the mass spectrometry data with public databases, we determined that, among the detected carotenoids, the Xanthophyll lutein (C_40_H_56_O_2_) was detected in all samples, but its content varied (0.0082–0.021 µg/g) (Figure 1E and Table S3).

In both blue peafowls and green peafowls, the Xanthophyll lutein content in the colored feathers growing on the rump and the mantle feathers was significantly higher than that in the non-colored breast feathers (*p* < 0.05) (Figure 1E). Specifically, the iridescent train feathers growing on the rump of the blue peafowl had the highest Xanthophyll lutein content, approximately twice that of the non-iridescent breast feathers; followed by the blue peafowl mantle feathers, with a Xanthophyll lutein content approximately 1.5 times that of the non-iridescent chest feathers. In green peafowls, although the Xanthophyll lutein content in the colored feathers growing on the rump and the mantle feathers did not differ significantly (*p* > 0.05) (Figure 1E), it was more than 1.5 times that of the non-iridescent chest feathers.

After dividing the ocelli area of the colored train feathers growing on the rump of the blue peafowl into four different color regions, we obtained more detailed results. The blue region of the train feather (Rump_B) had the highest Xanthophyll lutein content, significantly higher than the other color regions (*p* < 0.05). The purple region of the train feather (Rump_G) had extremely low Xanthophyll lutein content, even lower than that of the non-iridescent chest feathers.

### 3.2. Transcriptome Sequencing and Differential Gene Expression Profiling

**Figure 2:**
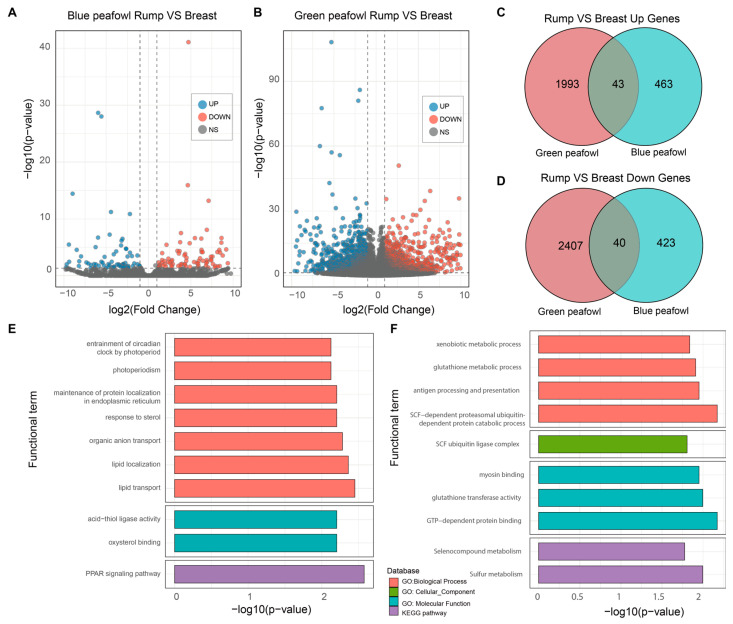
Differential gene expression analysis of feather follicle tissues from the peafowl’s rump and breast. Volcano plots show the differential gene expression analysis of the rump and breast feather follicle tissues of blue peafowls (**A**) and green peafowls (**B**). Genes with *p* < 0.05 and |log2 Fold Change| > 1 are considered differentially expressed genes. (**C**) Venn diagram showing the number of genes upregulated in the rump compared to the breast in blue peafowls and green peafowls. (**D**) Venn diagram showing the number of genes downregulated in the rump compared to the breast in blue peafowls and green peafowls. (**E**) Bar charts showing KEGG and GO enrichment analysis of genes that are upregulated in the rump feather follicle tissues compared to breast feather follicle tissues of blue peafowls and green peafowls. (**F**) Bar charts showing KEGG and GO enrichment analysis of genes that are downregulated in the rump feather follicle tissues compared to breast feather follicle tissues of blue peafowls and green peafowls.

To find the different gene expressions between the different feather follicle tissues, we constructed three RNA libraries (rump, mantle and breast) in blue peafowl and green peafowl. After sequencing and filtering, a total of 160.43 Gb clean reads were used to map to the peafowl genome. The summary of the mapping results is shown in Table S2. With the improvement of the annotation file, a total of 37,504 genes were identified. Considering that the most distinct coloration was observed in the breast and rump feathers, differential expression analysis was primarily focused on these two tissues to identify key regulatory genes. In blue peafowls, the breast feather follicle tissue exhibited 506 upregulated and 436 downregulated genes relative to the rump feather follicle tissue. In green peafowls, 2447 genes were upregulated, and 2036 genes were downregulated in the breast feather follicle tissue compared with the rump feather follicle tissue (Figure 2A). After taking the intersection of the upregulated and downregulated genes in the two types of peafowls, 39 upregulated genes and 41 downregulated genes were obtained. Among these, the upregulated genes detected in both types of peafowls were enriched in 9 GO terms and 1 KEGG pathway (Figure 2B). The downregulated genes were enriched in 8 GO terms and 2 KEGG pathways (Figure 2C–F). Notably, the PPAR signaling pathway KEGG pathway, in which the upregulated genes were enriched, is closely related to lipid metabolism.

### 3.3. Melanin and Xanthophyll Lutein Related Differential Gene Expression in Feather Follicle Tissue

**Figure 3:**
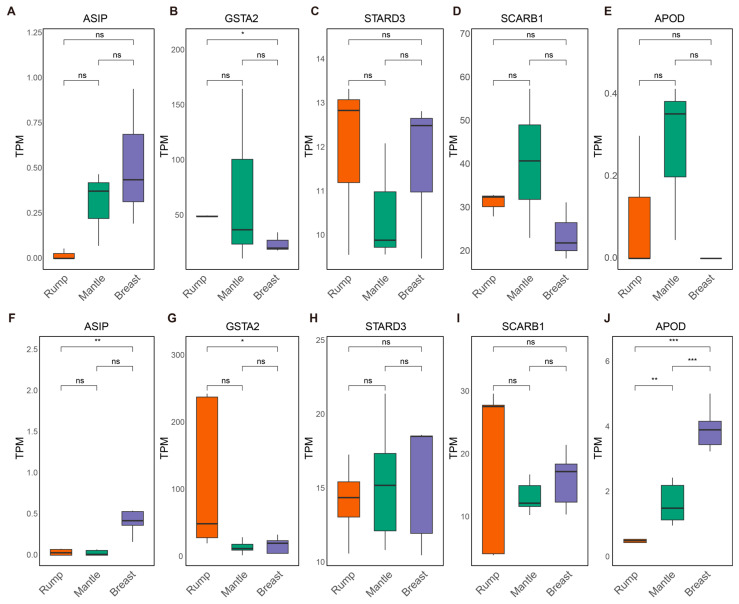
Expression of genes related to melanin and carotenoid deposition and transport in feather follicle tissues from different peafowl body parts. (**A**–**E**) show the expression levels of five genes in the green peafowl feather follicle tissues. (**F**–**J**) show the expression levels of five genes in the blue peafowl feather follicle tissues. Significant differences were determined by *t*-tests, and *p*-values were adjusted for multiple comparisons using the Benjamini–Hochberg (BH) method. Asterisks indicate significance based on adjusted *p*-values (^ns^ *p* > 0.05, * *p_adj_* < 0.05, ** *p_adj_* < 0.01, *** *p_adj_* < 0.001).

Among all differentially expressed genes (DEGs), the melanin synthesis-related gene *ASIP* (Agouti signaling protein) exhibited strikingly different expression patterns between rump feather follicle tissues and breast feather follicle tissues in both blue peafowls and green peafowls. Specifically, the expression level of *ASIP* in the rump feather follicle tissues of blue peafowls was less than one-thirtieth of that in the breast feather follicle tissues (log2FoldChange = 4.90, *p* < 0.05), while in green peafowls it was reduced to less than one-twelfth (log2FoldChange = 3.68, *p* < 0.05) (Figure 3A,F).

Furthermore, given that Xanthophyll lutein (one of the carotenoids) was detected in peafowl feathers and exhibited distinct distributions among differently colored regions, we examined the expression patterns of several carotenoid-related genes (*GSTA2*, *STARD3*, *SCARB1*, *APOD*) in different feather follicle tissues (Figure 3B–J) [30]. *GSTA2* (glutathione S-transferase alpha 2) is the closest avian homolog of *GSTP1*, a known carotenoid-binding protein in mammalian retinas, and has been implicated in carotenoid-based pigmentation in birds and fish [31,32]. Studies in fish species, such as *Botia dario*, have shown that *GSTA2* plays a key role in carotenoid deposition and scale coloration [33].

In this study, *GSTA2* expression was consistently higher in rump feather follicle tissues than in breast feather follicle tissues in both blue peafowls and green peafowls. Notably, in green peafowls, the expression level of *GSTA2* in rump feather follicle tissues was approximately three times higher than that in breast feather follicle tissues (log2FoldChange = −1.64, *p* < 0.05) (Figure 3B). In addition, we detected a significant difference in the expression of the carotenoid transport gene *APOD* between rump and breast feather follicles in green peafowls (log2FoldChange = −3.39, *p* < 0.05), whereas no such difference was observed in blue peafowls (Figure 3G). These results suggest potential interspecific differences in Xanthophyll regulation between blue peafowls and green peafowls.

## 4. Discussion

### 4.1. Structural Foundation and the Prerequisite of Melanin

The iridescent feather of the peafowl has long been regarded as a canonical example of structural coloration [1]. Previous studies based on scanning electron microscopy (SEM) and optical modeling have revealed that the cortex of peafowl feather barbules contains a two-dimensional (2D) photonic crystal structure composed of melanin rods embedded in a keratin matrix [2,34,35]. Furthermore, the different colored regions (blue, green, yellow, brown) on the train feathers growing on the rump are primarily controlled by geometric parameters, particularly the lattice constant, the number of periods, and the thickness of the keratin cortex [9]. Our previous research on white peafowls also demonstrated that melanin is a prerequisite for the production of feather coloration in peafowls [3]. In this study, we observed significant differential expression of the *ASIP* gene between feather follicles of the rump and breast. *ASIP* is a key regulatory factor in the pigmentation of animal skin, hair, and bird feather follicle tissues. It antagonizes melanocortin-stimulating hormone (α-MSH) signaling by binding to melanocortin receptor 1 (*MC1R*), thereby inhibiting eumelanin synthesis and promoting pheomelanin production [36,37,38].

Given that lower *ASIP* expression correlates with increased eumelanin deposition and darker pigmentation in other vertebrates, the relatively lower expression observed in peafowl rump follicles suggests a physiological environment conducive to high melanin synthesis [1,3,8,36]. This aligns with the observation that melanin rods are the fundamental building blocks of the photonic lattice. Without these rods—as seen in leucistic or albino variants—the structural basis for iridescence collapses, resulting in white feathers due to incoherent scattering [3]. Thus, the downregulation of *ASIP* in the rump may facilitate the melanin accumulation necessary for forming the functional 2D photonic crystal architecture. Conversely, insufficient expression of melanin-related genes would fail to produce the prerequisite structural foundation for peafowl iridescence.

### 4.2. The Overlooked Role of Xanthophyll Lutein Fine-Tuning Structural Color

While the structural contribution of melanin is well-established, previous optical models have predominantly focused on the interplay between melanin, keratin, and air channels, often treating the refractive indices of these components as the sole determinants of color [1,7,8,34,35]. These models typically attribute complex hues (like yellow and brown) to “mixed” structural colors resulting from varying lattice constants or reduced periodicities. However, the potential contribution of non-melanin pigments in iridescent regions has been largely underestimated.

Our study challenges the purely structural paradigm by identifying trace amounts of Xanthophyll lutein in peafowl feathers via high-sensitivity UPLC-MS/MS. The varying concentration of Xanthophyll lutein across different colored regions suggests that this carotenoid is not merely an antioxidant bystander but an active participant in coloration [11]. We propose that while the 2D photonic crystal generates the primary structural reflectance peak via interference, Xanthophyll lutein acts as a spectral filter, absorbing specific wavelengths to refine the hue and saturation. This explains why gene expression analysis revealed significant differences in the carotenoid deposition gene *GSTA2* between iridescent and non-iridescent follicles. Therefore, a complete model of peafowl coloration should integrate the physical scattering of the melanin-keratin lattice with the chemical absorption of Xanthophyll lutein.

We hypothesize that the intricate ocelli patterns are governed by the precise spatiotemporal regulation of genes involved in both melanin structure formation and carotenoid transport. The rare “black peafowl” variant provides a possible theoretical test case for this hypothesis. Future research should prioritize collecting samples from black peafowls to verify whether their phenotype results from the disruption of the photonic crystal structure itself or defects in the carotenoid transport pathway proposed in this study.

## 5. Conclusions

The discovery of lutein in peafowl feathers suggests that their iridescence is jointly constructed by structural and pigmentary coloration. Genetic analysis confirms that the differential expression of the melanin-related gene *ASIP* and the carotenoid-deposition gene *GSTA2* provides the hereditary foundation for this iridescence. This dual mechanism offers a new perspective on the evolution of optical signaling in birds.

## Data Availability

The raw RNA sequence was deposited at NCBI with BioProject accession number PRJNA1289077.

## Supplementary Materials

The following supporting information can be downloaded at https://www.mdpi.com/article/10.3390/ani16060903/s1. Table S1: Feather and feather follicle tissues samples collection; Table S2: Statistics of transcriptome sequencing data; Table S3: Metabolite annotation at RT = 557.1 s detected by Orbitrap Exploris 120.

## Abbreviations

The following abbreviations are used in this manuscript:

RNA	Ribonucleic Acid
ASIP	Agouti Signaling Protein
GSTA2	Glutathione S-transferase Alpha 2
STARD3	StAR Related Lipid Transfer Domain Containing 3
SCARB1	Scavenger Receptor Class B Member 1
APOD	Apolipoprotein D
MC1R	Melanocortin 1 Receptor
