# Supplementary material for: Structural Coloration and Carotenoids Together Create the Vibrant Colors of Peafowl Feathers
Source: Animals (Basel). 2026 Mar 13;16(6):903. doi: 10.3390/ani16060903 (PMC13023350; doi:10.3390/ani16060903)
Supplement: Supplementary file 1 [file animals-16-00903-s001.zip › Supplementary_Materials.pdf]

Table S1. Feather and feather follicle tissues samples collection.

Species	Sample_number	Feather_sample_numbers	Feather_follicle_tissue_sample_numbers
Blue peafowl	BP-1	BP-1-rump; BP-1-rump-B; BP-1-rump-Br; BP-1-rump-G; BP-1-rump-Br	BP-1-rump
		BP-1-mantle	BP-1-mantle
		BP-1-breast	BP-1-breast
	BP-2	BP-2-rump; BP-2-rump-B; BP-2-rump-Br; BP-2-rump-G; BP-2-rump-Br	BP-2-rump
		BP-2-mantle	BP-2-mantle
		BP-2-breast	BP-2-breast
	BP-3	BP-3-rump; BP-3-rump-B; BP-3-rump-Br; BP-3-rump-G; BP-3-rump-Br	BP-3-rump
		BP-3-mantle	BP-3-mantle
		BP-3-breast	BP-3-breast
Green peafowl	GP-1	GP-1-rump	GP-1-rump-1; GP-1-rump-2
		GP-1-mantle	GP-1-mantle-1; GP-1-mantle-2
		GP-1-breast	GP-1-breast-1; GP-1-breast-2
	GP-2	GP-2-rump	GP-2-rump-1; GP-2-rump-2
		GP-2-mantle	GP-2-mantle-1; GP-2-mantle-2
		GP-2-breast	GP-2-breast-1; GP-2-breast-2
	GP-3	GP-3-rump	GP-3-rump-1; GP-3-rump-2
		GP-3-mantle	GP-3-mantle-1; GP-3-mantle-2
		GP-3-breast	GP-3-breast-1; GP-3-breast-2

Table S2. Statistics of transcriptome sequencing data.

Species	Blue peafowl			Green peafowl		
Feather follicle tissues	Breast	Mantle	Rump	Breast	Mantle	Rump
Total reads	62763575	62760980	62771925	31351209	31405981.5	31374052.5
Total mapped reads	4614378034	4520673389	4547198247	22011711	22312675	22222653
Total mapped rate (%)	73.52	72.03	72.44	70.19	71.03	70.86

Table S3. Metabolite annotation at RT = 557.1 s detected by Orbitrap Exploris 120.

MS2_name	MS2_score	level	MS1_name	MS1_ppm	mz	rt	Formula	type	HMDB
Xanthophyll	2.51	level2	NA	NA	568.4272	557.1	C40H56O2	POS	HMDB0303013
NA	NA	level4	4-Hydroxy-4-methylglutamate	24.96906667	178.1669	557.1	NA	POS	NA
NA	NA	level4	N6-Succinyl Adenosine	3.713375613	384.3257	557.1	NA	POS	NA
NA	NA	level4	2-(alpha-Hydroxyethyl)thiamine diphosphate	15.17661343	470.3813	557.1	NA	POS	NA
NA	NA	level4	NA	NA	569.4312	557.1	NA	POS	NA
NA	NA	level4	SN38 glucuronide	-20.01059929	569.5246	557.1	NA	POS	NA
NA	NA	level4	NA	NA	572.3997	557.1	NA	POS	NA
NA	NA	level4	Cyclochlorotine	-21.09677516	573.4334	557.1	NA	POS	NA
NA	NA	level4	NA	NA	577.4805	557.1	NA	POS	NA
NA	NA	level4	NA	NA	578.484	557.1	NA	POS	NA
NA	NA	level4	Rhamnetin 3-laminaribioside	-11.82839261	641.5432	557.1	NA	POS	NA
NA	NA	level4	NA	NA	649.5041	557.1	NA	POS	NA
NA	NA	level4	NA	NA	657.483	557.1	NA	POS	NA
NA	NA	level4	NA	NA	722.559	557.1	NA	POS	NA
NA	NA	level4	NA	NA	876.634	557.1	NA	POS	NA
NA	NA	level4	NA	NA	83.8587	557.1	NA	NEG	NA
NA	NA	level4	Avenanthramide P	-2.598594046	354.3378	557.1	NA	NEG	NA
NA	NA	level4	Catechin 7-sulfate	-8.164069884	369.3197	557.1	NA	NEG	NA
NA	NA	level4	NA	NA	405.2951	557.1	NA	NEG	NA
NA	NA	level4	NA	NA	458.2893	557.1	NA	NEG	NA
NA	NA	level4	NA	NA	523.2607	557.1	NA	NEG	NA

NA	NA	level4	NA	NA	524.2651	557.1	NA	NEG	NA
NA	NA	level4	NA	NA	525.2595	557.1	NA	NEG	NA
NA	NA	level4	Dotmp	23.11986708	547.3044	557.1	NA	NEG	NA
NA	NA	level4	NA	NA	551.2858	557.1	NA	NEG	NA
NA	NA	level4	NA	NA	672.4985	557.1	NA	NEG	NA
NA	NA	level4	NA	NA	675.5147	557.1	NA	NEG	NA
NA	NA	level4	NA	NA	1021.8299	557.1	NA	NEG	NA
NA	NA	level4	NA	NA	85.0776	557.1	NA	POS	NA
NA	NA	level4	Ethyl 4-nitrophenyl methylphosphonate	10.29240816	246.1808	557.1	NA	POS	NA
NA	NA	level4	NA	NA	247.1843	557.1	NA	POS	NA
NA	NA	level4	Fluoroazomycin arabinoside	-10.01933798	248.1868	557.1	NA	POS	NA
NA	NA	level4	NA	NA	286.0329	557.1	NA	POS	NA
NA	NA	level4	NA	NA	366.1398	557.1	NA	POS	NA
NA	NA	level4	NA	NA	464.1187	557.1	NA	POS	NA
NA	NA	level4	QUINOLONE DERIVATIVE	-13.89039051	604.0489	557.1	NA	POS	NA
NA	NA	level4	NA	NA	228.9396	557.1	NA	NEG	NA
NA	NA	level4	NA	NA	287.9538	557.1	NA	NEG	NA

* Metabolites detected at a retention time (RT) of 557.1 s using an Orbitrap Exploris 120 mass spectrometer are listed. Compound annotation was performed based on accurate mass matching at the MS1 level and, when available, MS/MS spectral matching at the MS2 level. The MS2 score represents the similarity between experimental and reference fragmentation spectra. Identification confidence is reported according to commonly accepted metabolite annotation levels, where Level 2 indicates putative identification based on MS/MS spectral similarity and Level 4 indicates unknown or putatively characterized compounds based on accurate mass only. The mass error is expressed as parts per million (ppm). Both positive (POS) and negative (NEG) ionization modes are shown. Molecular formulas and Human Metabolome Database (HMDB) accession numbers are provided when available.
